# Treadmill Measurement of Maximal Aerobic Capacity in Untrained Students Aged 9–18 Year

**Published:** 2017-05

**Authors:** Yu Qiao MA, Yan Lin CHEN, Xiao Fang LI, Chen Yang BIAN, Li Na TANG, Si Jin MENG, Yi Zhen YU

**Affiliations:** 1. Dept. of Child, Adolescence and Woman Health Care, School of Public Health, Tongji Medical College, Huazhong University of Science and Technology, Wuhan, P.R. China; 2. Exercise Physiology Laboratory, Wuhan Institute of Physical Education, Wuhan, P.R. China

## Dear Editor-in-Chief

As an important function index to evaluate physical healthy, maximal aerobic capacity, does not only determine performance in a wide range of activities, but also a health-related parameter (1). Lower aerobic capacity is a strong predictor for a variety of disease and all causes of death in adults (2) and higher levels of aerobic fitness have been associated with superior cognitive control in children and across the lifespan (3). In general, aerobic fitness is measured by maximal oxygen consumption (VO_2max_) and VO_2max_ is considered the gold standard for measurement of aerobic fitness (4).

Childhood and adolescence are very important periods during which multiple physiological changes occur. Date obtained from the Chinese National Survey on Student’s Constitution and Health indicated that the levels of physical fitness had no significant improvement, although the anthropometric parameters presented an overall positive secular trend (5), and what worry was that physical fitness and cardiopulmonary function showed a sustained downward trend. Though China started relative late, domestic scholars also have done many researches in this field. However, the study systematically reported the maximal oxygen consumption for Chinese children and adolescents was conducted in 1995 (6) and there were few reports of maximal oxygen uptake, directly measured, in healthy Chinese children. Research is requisite regarding further. The direct determination maximal oxygen consumption demands sophisticated instrumentation, laboratory time, trained personnel, and is not a practical measurement for large population groups. Accordingly, it is kind of a necessity to develop VO_2max_ prediction equation.

Totally, 102 students (50 boys and 52 girls) aged 9 to 18 yr achieved VO_2max_ criteria during the graded exercise test and were included in the study. All tests were conducted in the Exercise Physiology Laboratory at Wuhan Institute of Physical Education from September 2013 to December 2013. All of the participants were Han Chinese, non-obese, healthy and taking no medications and none were in regular training programs of sufficient intensity.

The institutional Ethics Committee approved the experimental design and consent forms. Each student and his/her parents provided written informed consent.

The graded exercise test was performed on a computer-controlled treadmill (Pulsar 4.0, HP-Cosmos Sports & Medical, Nussdorf-Traunstein, Germany) and VO_2max_ was measured using Metalyzer II portable (Cortex Metalyzer II, Leipzig, Germany). SPSS version 18.0, (Chicago, IL, USA) was used to analyze the data.

The present Wuhan subjects had larger VO_2max_. As a whole, almost linear increases both in boys’ and girls’ VO_2max_ (measured in liters per minute) with age during adolescence and boys’ values were significantly higher than the girls’ at each age group, consistent with previous studies (7). Stepwise regression analysis showed that after adjusted for age and sex, the body fat, vital capacity and resting heart rate were three predictive variables selected in the best regression model (adjusted R2=0.833, *P*<0.001, F=95.733) and the obtained regression equation was as follows: VO2max=0.052×lean body mass−0.01×resting heart rate+0.009×vital capacity+0.767. In conclusion, overall, an almost linear increases both in boys’ and girls’ VO2max (measured in liters per minute) with age during adolescence and boys’ values were significantly higher than the girls’ at each age group, consistent with previous studies. When establishing the model, the prediction model revealed LBM, RHR and VC as explanatory variables of VO2max. Our study is only a cross-sectional study, so a large-scale research should make a longitudinal approach to explore more change rules of aerobic fitness with growth and age in future. At the same time, there is a need for further study in order to improve the accuracy in the prediction of VO2max.
